# Scrutinizing Social Identity Theory in Corporate Social Responsibility: An Experimental Investigation

**DOI:** 10.3389/fpsyg.2020.580620

**Published:** 2020-12-14

**Authors:** Agnieszka Paruzel, Martin Danel, Günter W. Maier

**Affiliations:** Work and Organizational Psychology, Department of Psychology, Bielefeld University, Bielefeld, Germany

**Keywords:** corporate social responsibility, social identity theory, organizational identification, job satisfaction, organizational commitment, organizational citizenship behavior

## Abstract

Corporate social responsibility (CSR) is widely established by companies that aim to contribute to society and minimize their negative impact on the environment. In CSR research, employees’ reactions to CSR have extensively been researched. Social identity theory is often used as a theoretical background to explain the relationship between CSR and employee-related outcomes, but until now, a sound empirical examination is lacking, and causality remains unclear. CSR can unfold its effect mainly because of three theoretically important aspects of CSR initiatives, which increase identification, i.e., distinctiveness, prestige, and salience of the out-group. This study examines how far identification can explain the effect of CSR on employees. In an experimental vignette study (*N* = 136 employees), CSR was manipulated in three degrees (positive, neutral, and negative) to examine its effects on job satisfaction, organizational commitment, and organizational citizenship behavior (OCB). In the vignettes, information on distinctiveness, prestige, and salience of the out-group were presented. Regression analyses showed that CSR significantly predicted commitment and job satisfaction, but not OCB. We found mediation effects of CSR on commitment, job satisfaction, and OCB through identification, but the effect of CSR on identification explained only little variance which indicates additional underlying mechanisms. The applicability of social identity theory for explaining CSR is discussed. Moreover, we discuss further explaining mechanisms.

## Introduction

Imagine Mary and John, both working in textile companies. Mary is working for a company that reduces its negative impact on the environment. From flyers and posters at work and local newspapers, Mary knows much about the company’s volunteer programs, which cover not only regional projects but also include educational programs in the production countries. Mary supports ethical production – she knows that the company pays all workers abroad fairly and that they work under safe conditions. John also reads about his company in the newspapers, but his company is blamed for irresponsible behavior. He is convinced that his company’s main strategy is to make more and more profit by saving unnecessary costs, often with negative environmental side effects and promoting social inequality. While Mary is satisfied with her company’s strategy, John is questioning the business practice of his employer, thinking about accidents in the production countries and the devastating environmental impact. Whose identification with their job and company is stronger?

Nowadays, employees become increasingly aware of the actions and policies that companies undertake to support local communities and common welfare (Raub and Blunschi, 2014), which is termed corporate social responsibility (CSR). CSR describes “context-specific organizational actions and policies that take into account stakeholders’ expectations and the triple bottom line of economic, social, and environmental performance” (Aguinis, 2011, p. 855). For a long time, CSR research focused on the financial advantages and increase in reputation associated with CSR, before individual reactions gained wide attention (Aguinis and Glavas, 2012). CSR can either be internal, which means that it focuses on internal stakeholder groups of CSR such as employees, or external, which means that it is focused on stakeholder groups outside of the company, such as customers or the natural environment and society in general (Rupp and Mallory, 2015; Glavas, 2016). CSR initiatives cover diversity policies and practices, ethical labor practices, employee training, philanthropic giving, community development programs, volunteerism initiatives, and environmental sustainability programs (Rupp and Mallory, 2015). The relationships between CSR and employees’ attitudes and behavior are well researched, and CSR has proven to affect job satisfaction, organizational commitment, and organizational citizenship behavior (OCB) in a positive way (Rupp and Mallory, 2015; Glavas, 2016; Gond et al., 2017). To sum it up, the society, in general, the company itself, and the employees may benefit from CSR. This study focuses on employees because, from a company perspective, they are the most important stakeholders regarding CSR and they are often planning, witnessing, and participating in CSR (Rupp and Mallory, 2015).

Despite numerous studies investigating the benefits of CSR for employees such as increased commitment and job satisfaction (Rupp and Mallory, 2015; Gond et al., 2017), less attention has been paid to the mechanisms of how these positive effects on employees occur. Experimental research on mediating mechanisms of CSR effects on employees is lacking (Glavas, 2016; Gond et al., 2017) and little is known about what exactly leads employees to be more satisfied with their job or be more committed to their employer when the company is socially responsible. This can only be achieved using experimental research designs allowing for causal interpretations. The present study sheds light on this black box. Social identity theory is the most frequently used theoretical framework to explain how CSR affects employees in a favorable way (Gond et al., 2017), but this theory has not sufficiently been investigated experimentally yet. Especially research is lacking which incorporates all theoretical assumptions stemming from social identity theory in the organization (Ashforth and Mael, 1989). The aim of this study is to investigate organizational identification as an explanatory mechanism in the context of CSR and employees’ attitudes and behavior using an experimental design, following the strong demands for experimental research on CSR (Aguinis and Glavas, 2012). We use social identity theory in organizations as a theoretical ground and contribute to the literature by illuminating causal relationships of CSR on organizational commitment, job satisfaction, and OCB. This thorough theory application allows us to discuss the applicability of organizational identification for explaining how CSR affects employees. Using experimental vignette methodology, both internal and external validity is enhanced (Aguinis and Bradley, 2014). Based on the knowledge of the underlying mechanisms, we learn about how organizations should communicate CSR initiatives internally to employees so that the postulated positive effects of CSR can unfold.

### CSR and Its Relationship With Employee-Related Outcomes

Analyzing CSR on the individual level is termed micro-CSR and includes investigating the effects of CSR on employees or other individuals. It is well known that employees’ perception of CSR is highly positively related to many beneficial outcomes, such as identification, commitment, job satisfaction, OCB, engagement, and intentions to stay (for reviews, see e.g., Aguinis and Glavas, 2012; Rupp and Mallory, 2015; Glavas, 2016; Gond et al., 2017).

Several studies, except the vignette study by Raub (2017; study 2), investigated the relationship between CSR and commitment, which describes “an emotional attachment to, identification with, and involvement in the organization” (Meyer and Allen, 1991, p. 67), in a correlational design (Turker, 2009; Farooq et al., 2014). Organizational commitment involves affective, normative, and continuous components, while the affective component is most researched. In spite of identification being part of the definition of commitment, identification and commitment are distinct concepts, because employees integrate the organization’s values into their self-concept when they identify with their organization, which is not the case for commitment (Riketta, 2005; Ashforth et al., 2008). When employees witness their companies engaging in social and environmental causes, they perceive that the company has high ethical values and, in consequence, they are more likely to feel committed to the company they are working for (Kim et al., 2010).

Job satisfaction – a pleasurable or positive emotional state resulting from the appraisal of one’s job or job experiences (Locke, 1976) – was also often investigated as an outcome of CSR, but to our knowledge only in cross-sectional research designs (Valentine and Fleischman, 2007; Shin et al., 2016). Job satisfaction depends on how employees evaluate their job situation (van Dick et al., 2004; Shin et al., 2016), which also includes the perception of CSR initiatives. A positive evaluation of CSR initiatives can increase job satisfaction.

Corporate social responsibility is associated with an increase in OCB, which is defined as “individual behavior that is discretionary, not directly or explicitly recognized by the formal reward system, and in the aggregate promotes the efficient and effective functioning of the organization” (Organ, 1988, p. 4), e.g., helping an over-strained colleague. When employees perceive that their company acts in line with their ethical values, they are inclined to behave according to these values as part of this company (Ellemers et al., 1999). They are likely to show positive behavior such as OCB because the company serves as a good example and sets a behavioral guideline in terms of citizenship behavior (Lin et al., 2010). However, to our knowledge, this association was only investigated in cross-sectional research designs (Gao and He, 2017; van Dick et al., 2019).

Although the relationships between CSR and commitment, job satisfaction, and OCB have been investigated numerous times using correlational research designs, we do not know if CSR actually has a causal *effect* on these employee-related attitudes and behavior (Aguinis and Glavas, 2012; Jones and Rupp, 2017).

Building on an experimental research design, we derive the following hypothesis:

*Hypothesis* 1: Positive information about CSR including distinctiveness, prestige, and salience compared to negative or neutral information (including distinctiveness, prestige, and salience) leads to (a) increased commitment, (b) increased job satisfaction, and (c) increased OCB.

### Organizational Identification

It remains unclear what exactly causes the effects of CSR on employees; there is no consensus among researchers regarding theory and mediating mechanisms (Rupp and Mallory, 2015; Gond et al., 2017), and theories lack strict experimental examination. The aim of this study is to examine how far identification can explain the effect of CSR on employees, as social identity theory is the most widespread and most important theory in individual-level CSR research (De Roeck et al., 2016; Gond et al., 2017). Although organizational identification is widely assumed as an explaining mechanism and identification has been investigated as a mediator several times (e.g., Farooq et al., 2014, 2017; Shin et al., 2016), the aspects of social identity theory leading to organizational identification have been overlooked so that we do not know for certain if the mechanisms underlying social identity theory can be transferred to micro-CSR.

Social identity theory (Tajfel, 1978; Tajfel and Turner, 1979) is a profound theory originally stemming from early research in social psychology. Concerning CSR, this means that employees perceiving CSR increase their identification with their company, which increases their commitment, job satisfaction, and OCB (e.g., Farooq et al., 2014, 2017; Shin et al., 2016). There is growing research interest in the construct of organizational identification because identification transforms the relationship of employees to their employers and results in increased work performance (for an overview, see Blader et al., 2017), health, and well-being (Jetten et al., 2017).

The basic principles of underlying social identity theory are (self-) categorizing processes. People categorize themselves and others as in-group and out-group members according to social attributes, such as gender, age, profession, or the quality of CSR initiatives. These categorizations create a feeling of belongingness. Even a mere (random) categorization into a group can create a feeling of belongingness as shown in studies using minimal group paradigm (Tajfel et al., 1971; Otten and Moskowitz, 2000). Working for a socially responsible company is a favorable social attribute and people strive for ethical companies as employers because positively valued group memberships enhance self-esteem (Ashforth and Mael, 1989). Organizational identification increases and, in turn, has favorable effects on further outcomes such as job satisfaction (Shin et al., 2016), commitment (Kim et al., 2010; Farooq et al., 2014), and OCB (Farooq et al., 2017). Perceiving internal CSR is associated with pride to be a member of the company, which in turn is linked to increased identification (Lythreatis et al., 2019).

According to Ashforth and Mael (1989), who applied social identity theory to the organizational context, three antecedents increase the tendency to identify with an organization: distinctiveness, prestige, and salience of the out-group (Ashforth et al., 2008). *Distinctiveness* is described as the uniqueness of a group and differentiates one group from another (Oakes and Turner, 1986). In the context of CSR, people are likely to identify with their company when the CSR initiatives are unique and stand out from other companies’ initiatives (Du et al., 2007). *Prestige* refers to the desire of people to identify themselves cognitively with winners (Ashforth and Mael, 1989) which enhances their self-esteem. Prestigious companies enjoy a good reputation because their CSR initiatives won awards or are reported in the media. *Salience* means how easily a group category or group membership comes into mind (Fitzsimmons, 2013). The awareness of other groups (salience of the out-group) increases the awareness of the in-group (Allen et al., 1983; Ashforth and Mael, 1989). Transferred to the context of CSR, salience describes the employees’ awareness of the CSR initiatives of other companies. In summary, employees are likely to benefit from CSR when they identify with the company they work for, which happens when its CSR initiatives are unique, when they are prestigious and enjoy a good reputation, and when employees are aware of the CSR initiatives and strategies of other companies. Some researchers tested single aspects of the social identity theory as mediators in the context of CSR, such as prestige (Kim et al., 2010; De Roeck and Delobbe, 2012; Farooq et al., 2017). For example, De Roeck et al. (2016) investigated prestige as a mediator in a three-wave longitudinal design. However, these three antecedents – distinctiveness, prestige, and salience of the out-group – have never been investigated simultaneously. Only incorporating all three aspects allows us to rigorously examine how far organizational identification explains the effect of CSR on employees.

Based on social identity theory, researchers have investigated how CSR affects commitment, job satisfaction, and OCB, which are among the most important employee-related consequences of CSR (Glavas, 2016). For the relationship between CSR and commitment, identification has been tested as a mediator next to trust in a cross-sectional design (Farooq et al., 2014). Identification was a stronger mediator than trust. According to social identity theory, positive CSR perceptions enhance organizational identification. This leads to the desire to maintain this positive identity and group membership, which translates into commitment. From this strong feeling of belongingness, employees derive satisfaction because it enhances their self-esteem (Ashforth and Mael, 1989). Using a two-wave longitudinal design, El Akremi et al. (2018) found that the relationship between CSR and job satisfaction was mediated by identification. Shin et al. (2016) investigated a sequential mediation model of CSR on job performance, sequentially mediated by identification and job satisfaction. Using a cross-sectional design, they found that when employees perceive that their organization engages in CSR, they are more likely to identify with their organization, which, in turn, translates into job satisfaction. For the relationship between CSR and OCB, research also indicates that identification has an important mediating role (Shen and Benson, 2016; Farooq et al., 2017).

Although identification has been tested as a mediator, experiments and research incorporating the three aspects that enhance organizational identification in parallel – distinctiveness, prestige, and salience (Ashforth and Mael, 1989; Ashforth et al., 2008) – are lacking. Testing identification as a mediator of the effect of CSR on employee-related attitudes and behavior is not sufficient to support social identity theory; rather, a closer look on the antecedents which are necessary for the formation of identification is needed. To test social identity theory in the context of CSR, and thereby assuming distinctiveness, prestige, and salience as critical to the formation of identification, we formulate the following hypothesis:

*Hypothesis* 2: The effect of CSR on (a) job satisfaction, (b) commitment, and (c) OCB is mediated by organizational identification.

## Materials and Methods

### Research Design and Procedure

In an experimental vignette study, CSR was manipulated in a within-subjects design to measure its effects on the dependent variables commitment, job satisfaction, and OCB. Vignettes are descriptions of situations aiming at manipulating different levels of independent variables (Aguinis and Bradley, 2014). The experimental vignette methodology can enhance both internal and external validity at the same time compared to usual experiments (Aguinis and Bradley, 2014) which face the dilemma of sacrificing external for internal validity (Scandura and Williams, 2000). Moreover, by presenting authentic scenarios, experimental realism is promoted, and independent variables can be manipulated. Therefore, the method allows for a causal interpretation of hypothesized effects (Aguinis and Bradley, 2014). Experimental vignettes are widely used in, e.g., leadership (Nübold et al., 2013; Marchiondo et al., 2015; Steffens et al., 2018), organizational justice (Ötting and Maier, 2018; Trinkner et al., 2019), and work design (Thompson et al., 2015; Zacher et al., 2017) studies.

In three experimental conditions, the vignettes contained either positive, neutral, or negative portrayals of CSR activities of a fictitious company serving as the manipulation of the independent variable. The CSR initiatives described in the vignettes were fictitious and designed based on social identity theory, by putting emphasis on the aspects that increase organizational identification according to the application of CSR on organizations by Ashforth and Mael (1989). This means that the initiatives were described as more or less distinct and prestigious and contained information on other companies’ CSR or not. This adds value to the literature, as former vignette studies in the field of CSR were not designed based on theoretical frameworks (Tsai and Yang, 2010; Evans and Davis, 2011; Kim and Park, 2011; Zhang and Gowan, 2012; Rupp et al., 2013; Jones et al., 2014). Each vignette contained the same introduction with general information on the described CSR initiative. As the study was conducted in Germany, the text was presented in German but is translated here (text in brackets indicates the structure and was not presented to the participants):

“You work for the company Elvoria GmbH [Ltd.] in the purchasing department. The company produces various products that are available in most hardware stores. Currently, employees have the opportunity to participate in a project focusing on social responsibility. All employees have been informed *via* e-mail and can apply for this voluntarily. You applied and are now part of the project team. The project team meets regularly to discuss ideas, topics, and implementation of projects that add value to society.”

In the positive condition, CSR initiatives were described as unique and generous in order to put emphasis on distinctiveness. *Prestige* was realized by describing the company as a winner of a highly prestigious national CSR award, and to manipulate salience of the out-group, another nearby and same-branch company’s CSR initiatives were described:

“[Distinctiveness] The company management initiated the project in order to get involved in social issues, because the company regards employees and society as two important pillars. The project members have already decided on special and innovative projects and are currently testing them. For example, early school leavers are to be given a chance at the company by offering longer-term, paid internships that offer the prospect of a career start. In addition, more environmentally friendly resources are to be used for production. [Prestige] Last year, the company was awarded the CSR Prize of the German Federal Government, a prize for special projects that demonstrate economic, ecological, and social responsibility. In addition, various national media reported positively on the project. [Salience of the out-group] Flexirea is the biggest competitor of Elvoria GmbH and offers its employees a similar project of this kind. At Flexirea, there is already disagreement between the project group and the management. The project work is not part of the working time here. In your company, Elvoria GmbH, sufficient capacity is freed up so that no additional work is required.”

In the neutral condition, *distinctiveness* was realized by describing the CSR initiatives as average and a medium level of prestige was realized by describing the company as nominated for a local unknown CSR award. Information on other companies’ CSR initiatives was given, but information on the location and branch was omitted so that they were perceived salient but less salient than in the positive condition:

“[Distinctiveness] The company management initiated the project in order to get more socially involved, because they hope that this will increase the company’s performance. The project members have already decided on some project ideas and are currently planning them. The first projects are already being implemented. For example, a project on the paperless office was initiated to reduce paper consumption in the company. In addition, resources are to be purchased and processed that barely meet the statutory environmental guidelines. [Prestige] Last year, the company was nominated for a regional award for innovative projects and finished in one of the bottom places. A colleague tells you that he has heard about the project but cannot give any further information. [Salience of the out-group] The company Flexirea offers its employees a similar project of this kind. Both companies have the same goal: to increase the company’s performance. Every quarter, there is a meeting for exchange between the two project groups to support each other. For example, the paperless office initiative is also implemented at Flexirea.”

In the negative condition, in order not to be perceived as distinct and unique, the company’s CSR initiatives were described as self-serving. To realize a lack of *prestige*, the company was described to be awarded with the Public Eye Award, an award given for purely profit-oriented globalization, indicating a negative reputation. No information on other companies was given so that other companies were not perceived salient at all; instead, further general information on the CSR initiatives were given to ensure that all vignettes had the same word count.

“[Distinctiveness] The company management initiated the project in order to gain a better reputation in the media, because recently, due to a serious accident in the company, profits dropped sharply. The project members have already decided on simple, easy, and not very original projects and are in the process of testing them. For example, unpaid internships are to be offered to school drop-outs, which will later be converted into temporary employment contracts. In addition, more cost-effective resources that are supposedly environment friendly are to be used. [Prestige] Last year, the company was awarded the Public Eye Award, an award for the most serious cases of human rights violations and environmental misconduct by companies. In addition, various national media reported negatively on the project. [Salience of the out-group] The project is scheduled for another 2 years and additional project members may join and leave over time. The application is open to all employees and they can apply at any time. A project membership runs for 6 months to give other employees a chance to be part of the project.”

Each vignette in the original German version counted 245 words to keep the manipulation degree constant in the three conditions. The vignettes were pretested in a sample of *N* = 26 students to ensure that the three aspects increasing organizational identification were manipulated as intended and the scenarios were perceived as realistic. For this purpose, participants reported how realistic they perceived the scenarios and filled in a manipulation check (see below). Based on the results, the neutral vignette was slightly adapted to achieve a more neutral level of CSR.

At the beginning of the experiment, participants were informed about the study and randomly assigned to one of the three conditions. Forty-six participants were assigned to the positive condition, 46 participants to the neutral, and 44 participants to the negative condition. Next, one of the vignettes was presented and the participants were instructed to imagine that they were working for the described company. To facilitate the participants’ imagination, the company was given a fictitious name and a logo was designed. In the following, participants filled in a questionnaire to assess the dependent variables, a manipulation check, and demographics. A university’s ethics committee approved the research design (file reference 1266).

### Measures

For all outcomes, the following instruction was given: “Please think about the described situation and imagine vividly you were in this situation right now. Rate the following statements as if you were an employee of this company. Please rate the probability of your agreement to the statements (1 = *very unlikely* and 5 = *very likely*, as well as 1 = *very unlikely* and 7 = *very likely*, respectively).” The sequence of the scales measuring the dependent variables was randomized.

Organizational identification, hypothesized as a mediating variable, was measured with six items by Mael and Ashforth (1992), used in the German translation (Kraus and Woschée, 2012). Responses were given on a 5-point Likert scale (*α* = 0.80). A sample item is “When someone criticizes this company, it feels like a personal insult.”

Affective commitment was measured using the German adaptation (Maier and Woschée, 2002) of the Organizational Commitment Questionnaire by Porter and Smith (1970). Fourteen items were responded on a 7-point Likert scale (*α* = 0.94). One item was omitted due to a strong conceptual overlap with the organizational identification scale. A sample item is “I talk up this organization to my friends as a great organization to work for.”

Job satisfaction was measured using a German short adaptation (Haarhaus, 2016) of the Job Descriptive Index by Smith et al. (1969). Only the subscales satisfaction with tasks, satisfaction with development opportunities, satisfaction with leadership, and general job satisfaction were used as the others were not appropriate with regard to the scenario described in the vignettes, e.g., the satisfaction with the colleagues. Twenty items were responded on a 5-point Likert scale (*α* = 0.95). A sample item is “My tasks are exciting.”

Organizational citizenship behavior was measured using the German translation (Staufenbiel and Hartz, 2000) of the OCB scale by Organ (1988). Twenty-five items were responded on a 7-point Likert scale (*α* = 0.84). A sample item is “I make constructive suggestions that can improve the operation of the company.”

For the manipulation check, participants had to indicate their perceptions of the distinctiveness, prestige, and salience of the out-group of the CSR initiatives described in the vignettes. Participants responded four items on 5-point Likert scales (*α* = 0.77). The following items were used: “The company is innovative and unique” for distinctiveness, “It seems that the described company has a low social status” for prestige (reverse-coded), “An exchange between the described company and other companies is taking place” for salience of the out-group as well as “The already launched CSR initiatives are significant contributions to the good of society” for an overall rating of CSR.

### Participants

The sample consisted of *N* = 136 German employees who were recruited online. The online experiment was completed by 155 participants, but five had to be excluded because of too many missing values. Fourteen participants were excluded based on a speed index (Leiner, 2019). They completed the survey three times as fast (under 4 min 20 s) as the average respondent did (*Mdn* = 14 min 27 s), so they could not have been able to read the vignette and items carefully (Breitsohl and Steidelmüller, 2018).

The final sample consisted of employees in the age between 21 and 63 years (*M* = 33.5, *SD* = 11.7), working 33.7 h a week on average (*SD* = 9.7, *Min* = 15, *Max* = 50). Fifty-six percent of the participants were female. Concerning education, 61% reported to have a university degree, and 49.3% had completed or were currently absolving a vocational education (multiple selection was allowed). Half of the participants were working in small and medium-sized enterprises (49.3%) with less than 250 employees, the other half worked in larger companies. Thirty-five percent of the participants reported that they had no experience with CSR (34.6%), 32.4% reported somewhat experience, 25.7% actively dealt with the subject of CSR, and 7.4% reported much experience or active participation in CSR.

## Results

The descriptive statistics and inter-correlations of the variables under investigation are displayed in Table 1. No unexpected correlations were noticeable.

**Table 1:** Summary of intercorrelations, means, and SD for the variables under investigation.

		*M*	*SD*	1	2	3	4	5	6	7	8	9
1	CSR	—	—	—								
2	Gender	—	—	0.03	—							
3	Age	33.45	11.75	−0.12	−0.10	—						
4	Work hours/week	33.73	9.68	0.06	−0.19	0.27^**^	—					
5	CSR experience	2.06	0.95	0.11	0.06	−0.12	−0.10	—				
6	Identification	3.44	0.76	0.20^*^	0.09	0.01	0.02	−0.06	*0.80*			
7	Commitment	4.54	1.21	0.62^***^	0.18^*^	−0.01	−0.06	0.11	0.36^**^	*0.94*		
8	Job satisfaction	3.62	0.73	0.55^***^	0.18^*^	−0.02	−0.04	0.09	0.34^***^	0.81^***^	*0.95*	
9	OCB	4.75	0.56	0.04	0.25^**^	0.22^*^	0.02	0.07	0.47^***^	0.24^**^	0.31^**^	*0.84*

*N* = 136. Values in Italic in diagonal are reliability coefficients. CSR was coded 1 = negative, 2 = neutral, 3 = positive. Gender was coded 1 = male, 2 = female.

* *p* < 0.05;

** *p* < 0.01;

*** *p* < 0.001.

### Manipulation Check

Results of the manipulation check indicate that CSR was successfully manipulated. First, we compared the means in the three conditions (Table 2). The distinctiveness of the CSR programs of the described company was perceived significantly different in the three conditions by the participants [*F* (2, 133) = 38.10, *p* < 0.001, *η* ^2^ = 0.36], as well as its prestige [*F* (2, 133) = 37.08, *p* < 0.001, *η* ^2^ = 0.36] and the salience of the out-group [*F* (2, 136) = 18.13, *p* < 0.001, *η* ^2^ = 0.22]. In addition, the general CSR rating differed in the three conditions [*F* (2, 133) = 24.41, *p* < 0.001, *η* ^2^ = 0.27]. Analysis of the means showed that the effects were shaped in the intended direction. As the hypotheses were tested using the macro PROCESS (Hayes, 2018) for the statistical software SPSS, and PROCESS does not include a coding system that allowed us to compare all three groups equally, we used ANOVA for the manipulation check.

**Table 2:** Means and SD for the items of the manipulation check.

		Distinctiveness		Prestige		Salience		General CSR	
	*N*	*M*	*SD*	*M*	*SD*	*M*	*SD*	*M*	*SD*
Positive	46	3.93	0.71	4.39	0.77	3.28	0.81	4.28	0.75
Neutral	46	3.35	0.97	3.37	1.14	3.91	0.95	3.87	1.11
Negative	44	2.32	0.96	2.36	1.37	2.77	0.89	2.84	1.12

Second, we tested if the variables of the manipulation check mediate the relationship between CSR and identification. A statistically significant mediation indicates that the manipulation was successful. We found confirming results for this assumption: the indirect effect was 0.246. The confidence interval did not include zero (95% *CI* = [0.069; 0.437]).

### Hypothesis Testing

To test the hypotheses, we used regression analysis with contrast coding of the independent variable (Cohen et al., 2013). This coding method allows for generating contrasts, which exactly represent the stated hypotheses. In our experiment, the three conditions, each representing either positive, neutral, or negative CSR, were contrast coded and represented by two contrast variables. The first contrast (C1) tested the positive condition against the negative and neutral conditions simultaneously, and the second contrast (C2) tested the neutral condition against the negative.

Contrast coding of a variable with three levels requires the generation of two contrasts (Cohen et al., 2013). Yet, to answer our hypothesis, we needed to interpret only the first contrast. For the sake of completeness, we will briefly report the results of C2 – the comparison of the neutral and negative conditions.

To test the hypotheses, we computed a mediation analysis using the macro PROCESS for SPSS by Hayes (2018) with a categorical independent variable (PROCESS version 3.1, SPSS version 25). The option Helmert coding allowed us to test the above-mentioned contrasts and we used model 4 to test mediation. The first contrast C1 follows the logic of comparing one group simultaneously to two other groups. The paths from CSR to the three independent variables job satisfaction (H1a), commitment (H1b), and OCB (H1c) in the mediation model, labeled as a total effect, tested Hypotheses 1a–c. In Tables 3–5, results are displayed. Interpreting C1, results showed that positive CSR information leads to significantly more job satisfaction and commitment as compared to negative and neutral CSR information but has no effect on OCB. Hypotheses 1a (commitment) and 1b (job satisfaction) can be supported, but Hypothesis 1c has to be rejected (OCB). Moreover, we found that concerning C2, neutral CSR information leads to more job satisfaction and commitment than negative CSR information, but not OCB.

**Table 3:** Mediation analyses of the effects of information about CSR (C1) on commitment, mediated by identification.

Commitment	Consequent									
		Y Commitment			M Identification			Y Commitment		
Antecedent		*B*	*SE*	*p*	*B*	*SE*	*p*	*B*	*SE*	*p*
X CSR	C1	1.27	0.17	0.000	0.29	0.14	0.032	1.15	0.17	0.000
	C2	1.14	0.20	0.000	0.14	0.16	0.387	1.08	0.19	0.000
M Identification		—	—	—	—	—	—	0.40	0.10	0.000
Constant		−4.52	0.08	0.000	−3.44	0.06	0.000	−3.16	0.37	0.000
		*R* ^2^ = 0.39			*R* ^2^ = 0.04			*R* ^2^ = 0.45		
		*F*(2,133) = 43.19, *p* < 0.001			*F*(2,133) = 2.69, *p* = 0.071			*F*(3,132) = 36.49, *p* < 0.001		
Indirect effect	C1							95% *CI* = [0.009; 0.283]		
	C2							95% *CI* = [−0.068; 0.216]		

*N* = 136. DV, dependent variable; B, unstandardized coefficient; CI, confidence interval. The contrast C1 tests the positive conditions against the neutral and negative conditions simultaneously, contrast C2 tests the neutral condition against the negative condition.

**Table 4:** Mediation analyses of the effects of information about CSR (C1) on job satisfaction, mediated by identification.

Job satisfaction	Consequent									
		Y Job satisfaction			M Identification			Y Job satisfaction		
Antecedent		*B*	*SE*	*p*	*B*	*SE*	*p*	*B*	*SE*	*p*
X CSR	C1	0.67	0.11	0.000	0.29	0.14	0.032	0.60	0.11	0.000
	C2	0.60	0.13	0.000	0.14	0.16	0.387	0.57	0.12	0.000
M Identification		—	—	—	—	—	—	0.24	0.07	0.000
Constant		3.61	0.05	0.000	−3.44	0.06	0.000	−2.79	0.24	0.000
		*R* ^2^ = 0.30			*R* ^2^ = 0.04			*R* ^2^ = 0.36		
		*F*(2,133) = 28.95, *p* < 0.001			*F*(2,133) = 2.69, *p* = 0.071			*F*(3,132) = 25.01, *p* < 0.001		
Indirect effect	C1							95% *CI* = [0.003; 0.180]		
	C2							95% *CI* = [−0.045; 0.129]		

*N* = 136. DV, dependent variable; B, unstandardized coefficient; CI, confidence interval. The contrast C1 tests the positive conditions against the neutral and negative conditions simultaneously, contrast C2 tests the neutral condition against the negative condition.

**Table 5:** Mediation analyses of the effects of information about CSR (C1) on OCB, mediated by identification.

OCB	Consequent									
		Y OCB			M Identification			Y OCB		
Antecedent		*B*	*SE*	*p*	*B*	*SE*	*p*	*B*	*SE*	*p*
X CSR	C1	0.06	0.10	0.547	0.29	0.14	0.032	−0.04	0.09	0.637
	C2	−0.03	0.12	0.826	0.14	0.16	0.387	−0.01	0.16	0.476
M Identification		—	—	—	—	—	—	−0.36	0.06	0.000
Constant		−4.75	0.05	0.000	−3.44	0.06	0.000	−3.52	0.20	0.000
		*R* ^2^ = 0.00			*R* ^2^ = 0.04			*R* ^2^ = 0.23		
		*F*(2,133) = 0.21, *p* = 0.812			*F*(2,133) = 2.69, *p* = 0.071			*F*(3,132) = 13.08, *p* < 0.001		
Indirect effect	C1							95% *CI* = [0.009; 0.249]		
	C2							95% *CI* = [−0.069; 0.162]		

*N* = 136. DV, dependent variable; B, unstandardized coefficient; CI, confidence interval. The contrast C1 tests the positive conditions against the neutral and negative conditions simultaneously, contrast C2 tests the neutral condition against the negative condition.

Concerning the mediation analyses, all paths of the mediations are displayed in summary in Tables 3–5. To test Hypotheses 2a-c, we rely on the confidence intervals of the relative indirect effects, which are the product of the effect of CSR on identification and the effect of identification on the outcome. We used PROCESS with 5,000 bootstrap samples to compute 95% confidence intervals for the relative indirect effects. If the confidence intervals do not include zero, the relative indirect effect is statistically significant and indicates mediation. Regarding C1, the effects of CSR on commitment, job satisfaction, and OCB, each mediated by identification, the confidence intervals for the indirect effects did not include zero, which means that the effects of CSR on commitment (H2a), job satisfaction (H2b), and OCB (H2c) were each mediated by identification (Table 3). In the case of OCB, the total effect of CSR on OCB and the direct effect of CSR and identification on OCB were not significant, but we found a significant indirect effect, indicating an “indirect-only mediation” (Zhao et al., 2010). In sum, the data support Hypotheses 2a–c. Moreover, concerning C2, we found no mediating effects of identification for the effect of CSR on the three outcomes commitment (Table 2), job satisfaction (Table 4), and OCB (Table 5). Noteworthy is that CSR explained only 4% of the variance when predicting identification.

## Discussion

In the workplace, employees not only perceive how their company treats their employees but they also observe the quality of their company’s relationships to other stakeholders. It has long been assumed that employees’ CSR perceptions have resulted in an increased identification with their company, which would lead to positive attitudes and behavior relevant in the workplace such as commitment, job satisfaction, and citizenship behavior. Research on CSR on individual-level CSR was dominated by correlational studies. Identification was assumed to explain why employees react to CSR but has not been sufficiently investigated. We investigated the role of organizational identification as an explaining mechanism for the effects of CSR on employees using an experimental vignette design. The results show that there is a direct influence of CSR perceptions on commitment and job satisfaction but not on OCB. Organizational identification mediates the relationships between CSR and the dependent variables’ commitment, job satisfaction, and OCB. This means that employees perceiving positive CSR in their company should identify more with their company. We interpret the results that employees not only consider themselves as members of their company but also incorporate their working place into their self-concepts (Sluss and Ashforth, 2008). This strong feeling of belongingness manifests in a feeling of commitment toward their company. Moreover, employees are satisfied with their job, because the values of their company are also rooted in their own self-concepts. Strong organizational identification also evokes OCB. Employees behave in line with the values of the company.

Despite no total effect of CSR on OCB, the relationship between CSR and OCB is mediated by identification. We explain the reason why there is no total effect with the concept of identity (Ashforth et al., 2008). It indicates that behavior is a more distal aspect of a person’s identity than their attitudes. Ashforth et al. (2008) distinguish the core, content, and behaviors of identity. Employees can not only think and feel their way into identification but also act their way into identification. Whereas the core of identity underlies a narrow definition of identity and contains self-definitions, importance as well as effect, content, and behaviors of identity are broader concepts and conceptually more distant from the core of identity. The content of identity includes values, goals, beliefs, stereotypic traits and knowledge, skills, and abilities. Commitment and job satisfaction fall into this category. Behaviors of identity are most distant from the core of identity; OCB falls in this category. Based on the distance between the core of identity and behaviors of identity, the authors regard behavior not as a “necessary component” of identity, but as a “probabilistic outcome of identification” (Ashforth et al., 2008, p. 311). As we found direct relationships between CSR and attitudinal outcomes such as commitment and job satisfaction, it is possible that CSR cannot directly evoke behaviors of identity such as OCB due to the distance to the core of identity. This is in line with the results of Evans et al. (2011). They investigated the relationships between perceived corporate citizenship (PCC – in other words, CSR) as the independent variable and identification and OCB as dependent variables. They found a positive significant relationship between PCC and identification. The relationship between PCC and OCB was only positive and significant for those people who are highly regarding value-oriented which means that they are not concerned only about themselves but also about others. In their study, they also did not find a direct relationship between CSR and OCB, and in their study, the relationship between CSR and OCB was only revealed when other aspects were considered. Other researchers also came to this conclusion: Identification transmitted the relationship between CSR and OCB only when the employees’ importance of and values toward CSR as a moderating factor was taken into account (van Dick et al., 2019).

Although our findings, specifically the tests of our hypothesis, provide evidence for organizational identification as an explaining mechanism for the effects of CSR on employees, the low variance explanation is attracting attention: CSR only explains 4% of the variance while predicting identification. Therefore, we cannot promote identification as the most important mediating mechanism with clear conscience any longer. The number of studies relying on social identity theory in the context of CSR does not indicate that it is the most important explanation of why CSR affects employees. Instead, we will demand the consideration of other mediators or more complex mediation models combining several mediating mechanisms in the future. In the following, we summarize the most prominent and often-cited theories in the following, which should be investigated in future research. A conjoint investigation will guide theory formation in the field of micro-CSR. Examples of other theory formation in the context of CSR are the concept of organizational justice, signaling theory, or meaningfulness (for an extensive review, see Rupp and Mallory, 2015).

From an organizational justice point of view, researchers argue that employee perceptions of the presence of CSR in their company can be regarded as third-party observations of organizational justice to the extent that the company acts fairly toward other stakeholders (Rupp, 2011). Consequently, employees infer that they will also be treated fairly in this company, which increases their satisfaction and commitment as well as increases OCB. Signaling theory (Rynes, 1991) relies on a similar principle: The presence of CSR serves as a positive signal so that employees and job applicants expect benefits for themselves from the organizations’ engagement in CSR, comparable to the halo effect (Nisbett and Wilson, 1977). In the context of CSR, this theory was investigated in an experimental setting as well as in a field study (Jones et al., 2014). Others argue that the perception of CSR produces meaningfulness (Seivwright and Unsworth, 2016; Aguinis and Glavas, 2019). CSR initiatives make work meaningful because employees – indirectly and in some cases even directly – contribute to a better society. Their personal values can unfold at work, which increases their satisfaction. They also want to give something back, which can possibly manifest in helping behavior toward their colleagues or OCB.

Finally, we promote further theory formation, integration, and strict experimental theory testing on the question of why CSR affects employees. This is especially important if theories are being transferred from other contexts. Up to now, theories do not make any statements about the negative effects of a low amount of CSR initiatives. This is illustrated by our findings concerning the comparison of neutral and negative CSR perceptions regarding their effects on commitment and OCB. Neutral CSR perceptions are associated with an increase in commitment and job satisfaction as compared to negative CSR perceptions. However, these effects are not mediated by identification, so that organizational identification seems to be appropriate as an explaining mechanism for only positive CSR perceptions. Participants could have perceived the CSR initiatives that are depicted in the negative vignette as corporate social irresponsibility (CSiR). However, research on CSiR is scarce, but Jones et al. (2009) theorize about CSiR and CSR as a continuum or a linear relationship. CSiR is the antithesis to CSR and accounts for the fact that companies may act irresponsibly under certain circumstances although they usually attach importance to CSR and act in a socially responsible way most of the time. CSiR is defined as “corporate actions that result in (potential) disadvantages and/or harm to other actors” (Lin-Hi and Müller, 2013, p. 1932). CSiR may involve a breach of law and can have disastrous consequences for the operating company (Jones et al., 2009; Lin-Hi and Müller, 2013). The concept helps companies to identify weaknesses and address those (Jones et al., 2009). Our vignettes do not likely depict CSiR, as the negative condition was not rated extremely bad concerning CSR (*M* = 2.84, *SD* = 1.12 on a 5-point scale; *N* = 44). Lin-Hi and Müller (2013) distinguish “doing good” and “avoiding bad” and assume that doing good is more effective than avoiding bad. Our results point in the same directions. However, we cannot conclude if the positive effect of CSR is stronger than the negative effect of CSiR because of the operationalization of the negative vignette. To sum it up, theory formation and investigation incorporating CSiR is a crucial point for future research, especially if the same psychological explaining mechanisms apply to CSiR as to CSR.

### Limitations and Recommendations for Future Research

Although our study can make several contributions to the literature, there are, however, some limitations. Vignette methodology studies are often criticized for threats to external validity (Scandura and Williams, 2000). However, by presenting realistic scenarios, experimental realism can be increased (Aguinis and Bradley, 2014). We reached this goal by a pretest of the vignettes and the manipulation check proves that manipulation of CSR was successful, so we can assume that the participants could put themselves in one of the three described situations and imagine it vividly. Vignette methodology is commonly used in, e.g., leadership (Nübold et al., 2013; Marchiondo et al., 2015; Steffens et al., 2018), organizational justice (Ötting and Maier, 2018; Trinkner et al., 2019), and work design research (Thompson et al., 2015; Zacher et al., 2017). In addition, the method enabled us to design the vignettes strictly according to the social identity theory and to incorporate information about distinctiveness, prestige, and salience, which has never been investigated before. Nevertheless, we would encourage researchers to investigate the effects of CSR on employees by means of intervention studies.

By using intervention studies, the communication strategy of CSR initiatives to employees can be manipulated according to different theoretical frameworks to subsequently measure the outcomes. We describe an example of an intervention study in the following. In experimental intervention studies, researchers measure the effects of an intervention and compare them to other interventions and/or a control group. A study that compares two interventions on CSR communication, would involve at least two companies. In one company, an article about CSR in the company newsletter can be written in a way that targets identification (by focusing on distinctiveness and prestige) whereas in another company the article could be written in a way that focuses third-party justice perceptions to measure its effects on employee-related attitudes and behavior. In this way, we can learn how employees react to CSR and compare identification and justice as explaining mechanisms.

Furthermore, generalizability is often a problem of experimental designs. However, the combination of realistic scenarios described in the vignettes and a population that consists of employed persons counteracts this threat.

### Recommendations for Practice

All theories on CSR and employee-related outcomes on the micro-level have in common that it depends on which CSR activities and policies the employees perceive, and not what the company actually and objectively does in terms of CSR (Rupp and Mallory, 2015). This also has to be considered when designing CSR initiatives. Employees will value no CSR initiative positively if they do not even know about it so that communication becomes an important success factor of CSR (Du et al., 2010). We recommend companies not only to community their CSR report on their website but also to focus on internal CSR communication by informing employees about CSR initiatives. As our study revealed a causal relationship, this will increase the employees’ job satisfaction and commitment. Internal communication can be achieved by a regular company e-mail newsletter, a printed newspaper, posters, and brochures, just to name a few. By including photos or info graphs, the employees get a better picture of their company’s CSR. A participative leadership style can also be favorable for perceptions of internal CSR (Lythreatis et al., 2019).

Moreover, the communication strategy could be designed according to the most effective explaining mechanism. Concerning identification, this would include incorporating “we-language” and phrases such as “moving the world together” (Hyundai Motor Group, 2020) to create a sense of belongingness. However, more research is needed concerning the most effective psychological mechanism.

### Conclusion

Our results show that employees react to CSR and that CSR affects their attitudes such as commitment and job satisfaction. We examined how far organizational identification can explain the effects of CSR on employees. Using an experimental vignette methodology design, we investigated causality and found that CSR has a rather strong causal influence on commitment and job satisfaction. Although identification mediated the effect of CSR on commitment, job satisfaction, and OCB, CSR explained only little variance of identification. This strongly indicates that there are further explaining mechanisms that should be considered. We would encourage researchers to investigate other theories in experimental settings and to include CSiR in future research. All in all, next to the widely investigated financial and reputation-related importance of CSR to companies in general, this research stresses the importance of CSR to employees.

## Data Availability Statement

The raw data supporting the conclusions of this article are available at https://osf.io/xd5nr/.

## Ethics Statement

The studies involving human participants were reviewed and approved by Ethics Committee of Bielefeld University (file reference 1266). Written informed consent for participation was not required for this study in accordance with the national legislation and the institutional requirements.

## Author Contributions

AP had the idea for this manuscript, crafted the first version of this manuscript, and revised it. AP, MD, and GM were involved in the design of the study. MD conducted the study and acquired participants. AP and MD performed the statistical analyses. GM supervised this work and critically reviewed the first draft. All authors contributed to the article and approved the submitted version.

### Conflict of Interest

The authors declare that the research was conducted in the absence of any commercial or financial relationships that could be construed as a potential conflict of interest.
